# Syndesmotic malreduction may decrease fixation stability: a biomechanical study

**DOI:** 10.1186/s13018-020-01584-y

**Published:** 2020-02-21

**Authors:** Lu Bai, Wentao Zhang, Siyao Guan, Jianxin Liu, Peng Chen

**Affiliations:** 1 Department of Sports Medicine, Peking University Shenzhen Hospital, #1120 Lianhua Road, Shenzhen, Guangdong Province China; 2 National and Local Joint Engineering Research Center of Orthopaedic Biomaterials, Peking University Shenzhen Hospital, #1120 Lianhua Road, Shenzhen, Guangdong Province China; 3 Department of Rehabilitation, Peking University Shenzhen Hospital, #1120 Lianhua Road, Shenzhen, Guangdong Province China

**Keywords:** Syndesmotic separation, Malreduction, Biomechanical stability

## Abstract

**Background:**

This study aims to investigate the malreduction of syndesmosis and its effects on stability.

**Methods:**

The biomechanical tests, including the three-dimensional (3D) displacement of the syndesmotic incisura, fibular rotation angle, and torque resistance, were performed on six cadaver legs. These specimens were first tested intact (intact group), then cut all the syndesmotic ligaments and fixed in anatomical position (anatomical model group) and test again. After that, syndesmosis was fixed in 1 cm malreduction (anterior and posterior displacement group) to do the same test.

**Results:**

In internal or external load, there were significant differences in torque resistance and fibular rotation angle (internal *t* = 2.412, *P* = 0.036; external *t* = 2.412, *P* = 0.039) between the intact and post-malreduction groups. In internal rotation load, there were significant differences in sagittal displacement between the intact and post-malreduction groups (*P* = 0.011), and between the anatomical and post-malreduction groups (*P* = 0.020). In external rotation load, significant differences existed between the intact and ant-malreduction group (*P* = 0.034) in sagittal (anterior-posterior) displacement. Significant differences also existed between the intact and post-malreduction groups (*P* = 0.013), and between the anatomical and post-malreduction groups (*P* = 0.038) in coronal (medial-lateral) displacement.

**Conclusions:**

Malreduction in different conditions does affect the stability of the syndesmotic fixation. The result of the study may reveal the biomechanical mechanism of poor clinical outcome in syndesmosis malreduction patients and pathological displacement patterns of the ankle under syndesmotic malreduction conditions.

**Level of evidence:**

III

## Background

Syndesmotic separation is a common injury associated with ankle fractures, and its incidence in fracture is approximately 7–20% [1, 2]. Anatomical reduction and rigid fixation of both the fracture and syndesmosis should be performed to acquire a good clinical result. However, it is difficult to accurately judge the reduction of the syndesmosis during surgery. Furthermore, due to the improper position of the syndesmotic screw or abnormal placement of the reduction forceps, syndesmosis may be fixed at the non-anatomical positon, which normally leads to malreduction. The concept of syndesmotic malreduction was reported by Gardner et al. in 2006 [3] after observing the bilateral computed tomography (CT) scans of 25 patients with ankle fractures and syndesmotic injury. They proposed that the distance between the anterior and posterior margin of the fibula and syndesmosis defines the malreduction. That is, when the measured ipsilateral and contralateral values differ by 2 mm (G value), this can be called a malreduction. In 77% (10/13) of patients with syndesmotic malreduction, the relative displacement and external rotation of the fibula relative to the tibia were the major persistent deformities. Sagi et al. [4] conducted a 2-year postoperative follow-up on syndesmotic malreduction patients, and the results revealed that the functional scores in the malreduction group were significantly lower than scores in the anatomical reduction group. However, the underlying mechanism that makes the difference remains unclear. Does syndesmotic malreduction cause mechanical instability that might affect ankle function? In order to further test this question, a biomechanical study was performed by directly investigating the mechanical relationship between syndesmotic malreduction and its stability.

## Materials and methods

### Storage and preparation of bone specimens

Six adult (age range 49–67 years old; four males and two females) legs (including the complete tibia and fibula, ankle, and feet) were included for this study, with the permission of the Anatomy Department of the Medical College of Shenzhen University. Radiographic examinations were performed to exclude pathological conditions (pre-exist trauma, neoplasm, bony deformity). The whole specimens were stored at − 20 °C prior to the mechanical experiments. Before the mechanical test, the entire specimens were thawed at room temperature for 12 h, and saline was sprayed during the thawing process to prevent the specimens from drying up.

### The establishment of testable models

Before the biomechanical experiment, all the muscles and tendons of the leg were removed, while the interosseous membrane and proximal/distal tibiofibular syndesmosis were kept intact (intact model). Soft tissues of the foot were left to enhance fixation into the machine. CT scans were performed to virtually make the 3D-printed guide template, and ensure that the bone tunnels of the syndesmotic fixation, including late anatomical fixation and malreduction fixation, do not interfere with each other. Then, all tibiofibular syndesmotic ligaments and the interosseous ligament were cut off to establish the syndesmotic separation model. A suture-button (Arthrex, Naples, USA) was used as the syndesmotic fixation (anatomical model). In order to establish the anterior malreduction model, the fibula was moved anteriorly by 1 cm, relative to the syndesmosis incisura (ant-malreduction model), or moved posteriorly by 1 cm to establish the post-malreduction model.

### Experimental protocol and specimen allocation

**Fig. 1:**
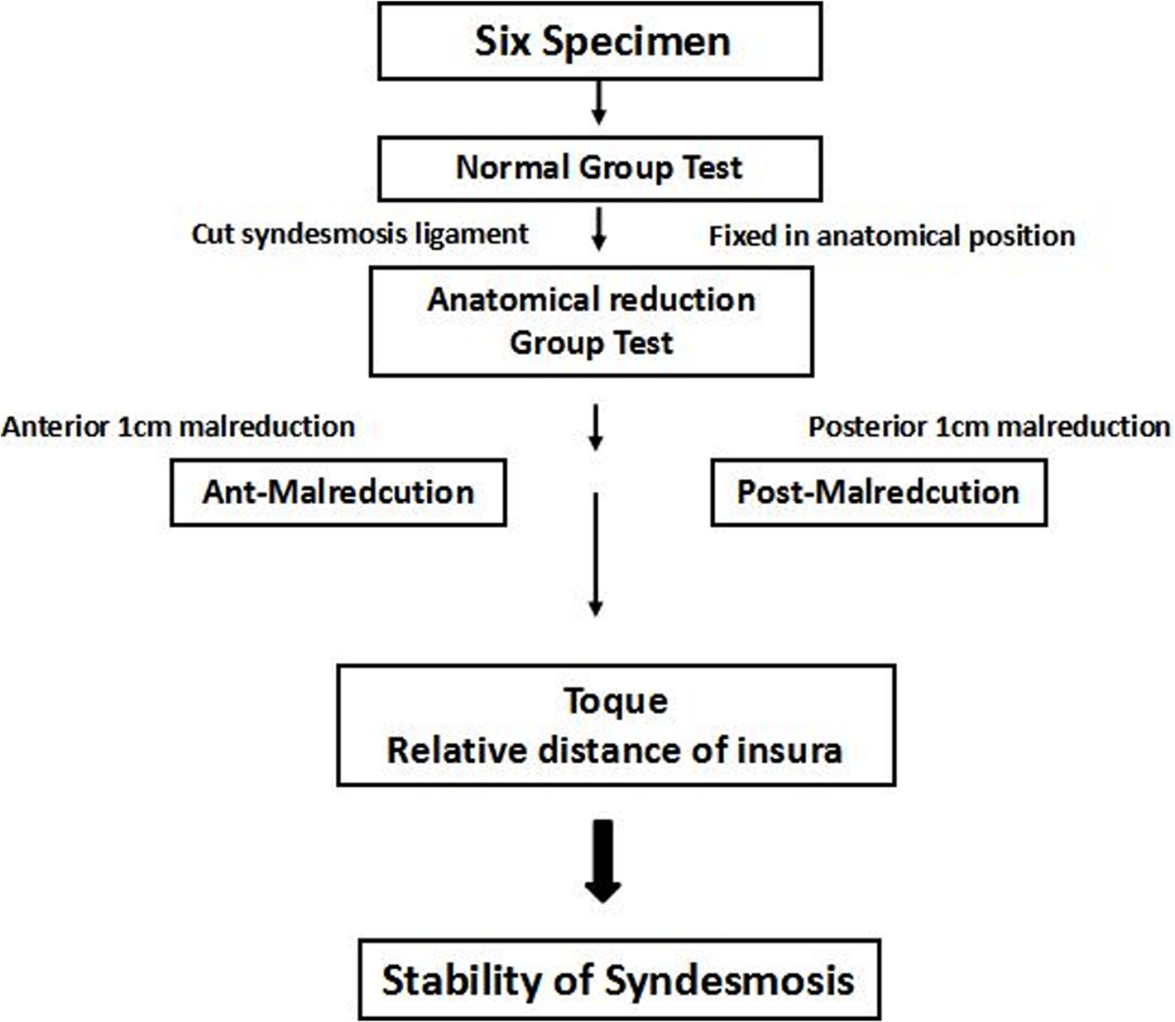
Experimental protocol

**Fig. 2:**
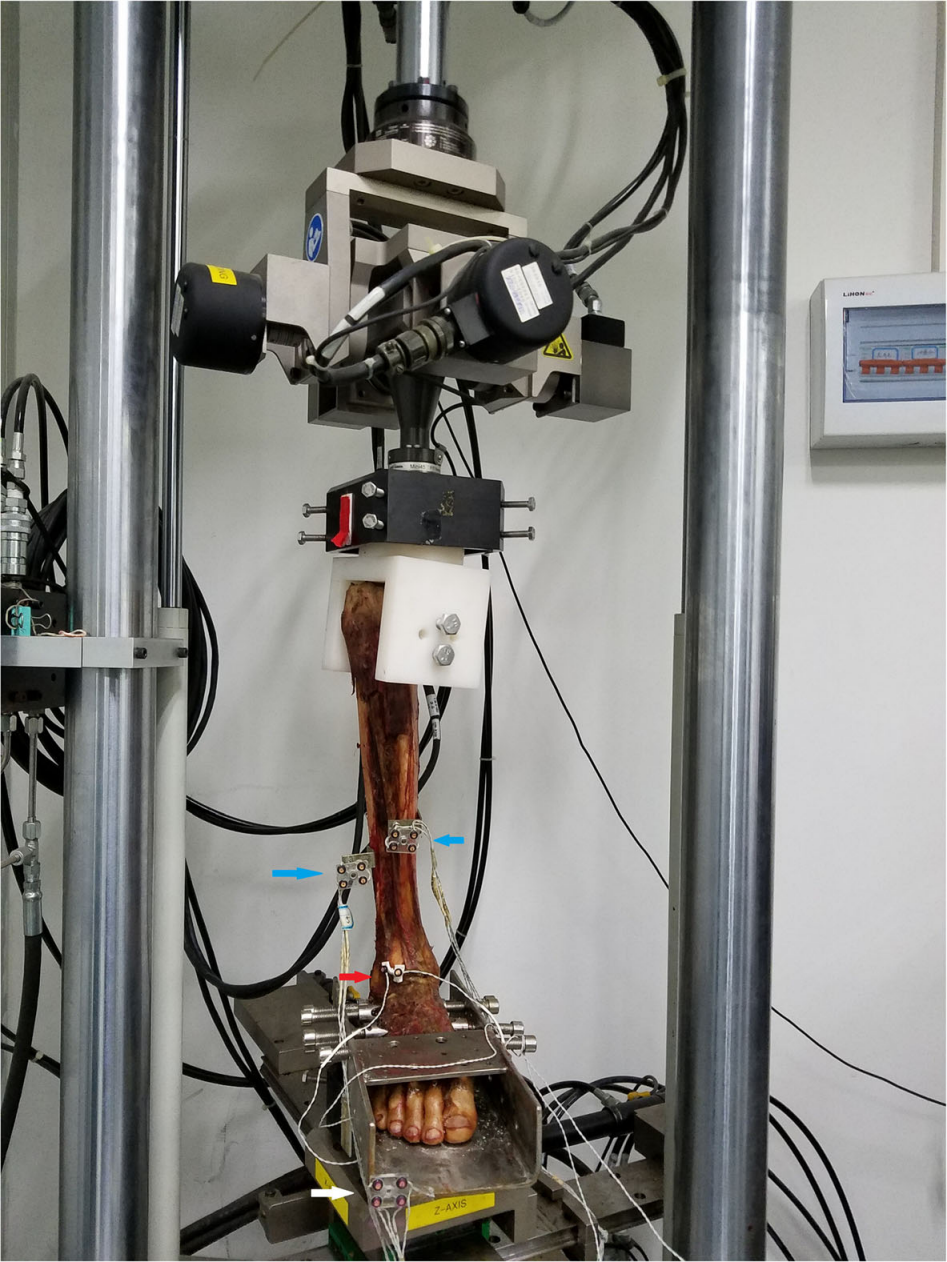
Experimental setup. The specimen was fixed on MTS machine. Blue arrow pointed optical marker of tibia and fibular. Red arrow pointed marker of syndesmosis incisura. White arrow pointed to the marker fixed on the base of the system

**Fig. 3:**
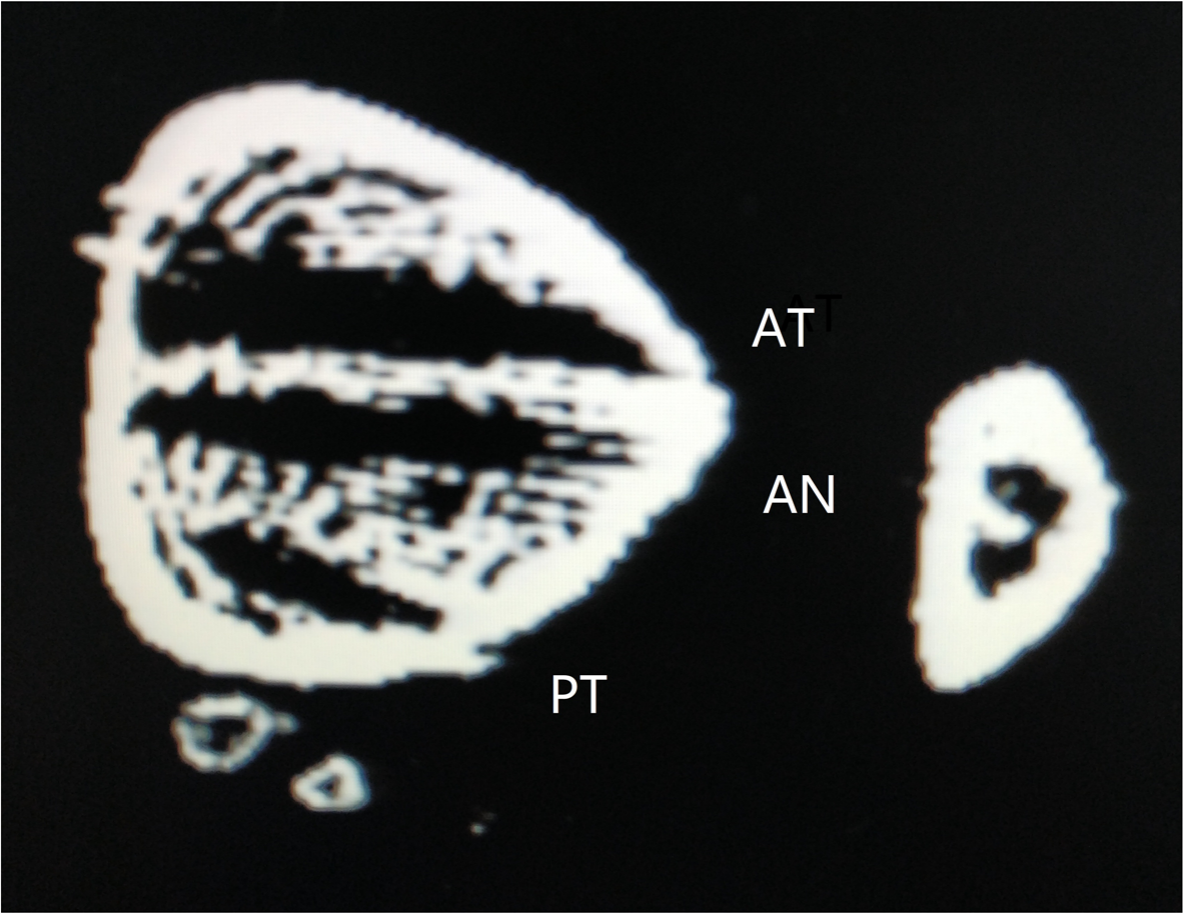
The CT scan of different bony tunnels created by 3D template. AT, anterior malreduction group; AN, anatomical group; PT, posterior malreduction group

1. Biomechanical tests were performed with all syndesmotic structures intact. These data was recorded as the *intact group*.
2. The ligaments of the syndesmosis were cut off, the 3D-printed template was used to make bone tunnels, and a suture-button was used to anatomically fix the syndesmosis. Afterwards, the same abovementioned biomechanical tests were performed, and these data were recorded as the *anatomical model group*.
3. With the lateral fibular bone tunnels unchanged, the fibula samples were moved anteriorly and posteriorly by 1 cm to establish the ant-malreduction and post-malreduction groups, respectively. Then, a 3D-printed guide plate was used to re-fix the specimens, in order to form the malreduction model (ant-malreduction and post-malreduction groups). Afterwards, the same biomechanical tests were performed, and data were collected as the ant-malreduction and post-malreduction groups. Figure 1 presents the experimental protocol, Fig. 2 shows the experimental setup, and Fig. 3 shows the CT scan of three bone tunnels made by template.

### Biomechanical testing

#### Testing apparatus

The biomechanical test was performed using an MTS simulator. The simulator included different channels, such as axial displacement (proximal-distal), sagittal displacement (anterior-posterior), coronal displacement (medial-lateral), and torsional rotation. Moreover, the system was equipped with a six degrees of freedom (6DOF) force transducer (ATI-Mini 45-SI-580-20, Schunk GmbH & Co. KG, Germany), which could record the Tx (axial), Ty (sagittal), and Tz (coronal) displacement.

#### Specimen fixation and marker placement

The proximal tibia of the specimens was fixed on the superior fixture. Then, the foot was rigidly fixed on the fixture, and the fixture was secured to the base via eight screws.

The Optotrak Certus motion analysis system (Northern Digital Inc., Ontario, Canada) was applied to capture the motion of the tibia and fibula. Four active optical markers were firmly fixed on the middle of the tibia and fibula, in order to set the base coordinate system. Another two active optical markers were placed on the incisura of the syndesmosis on the fibular side and tibial side, in order to record the initial coordinates of the two bone landmarks. All displacements and relative angles of the syndesmosis during the test were captured in this system. Before the test, all markers were captured at a sampling rate of 100 Hz for 5 s to record the initial relative position between the incisura edge of the syndesmosis in the tibial and fibular side. The whole set of the apparatus and detail were showed in Fig. 2.

#### Biomechanical test

We set the experimental conditions according to the method of previous literature [5, 6]. At the beginning of the test, the specimens were preconditioned for five cycles to ± 7.5 Nm, while a 600-N axial compressive load was applied and maintained. Subsequently, the specimens were tested in an intact, anatomical, and anterior/posterior malreduction state in sequence. For each state, the specimens were first placed in a neutrally positioned orientation without axial load. Then, a 600-N axial compressive load was applied to simulate the weight bearing load. While maintaining the axial compressive load, the foot was externally rotated to 10°, and internally rotated to 10° (1°/min). During the test, the coordinates of these two rigids, torque, and rotational degrees were continuously recorded. The Optotrak Certus motion analysis system can catch and record the micro-displacement of the syndesmosis markers in three-dimensional spaces.

### Statistical analysis

Statistical analysis was conducted in the PASW 18.0 software (IBM Chicago, USA). The metric data were reported as mean ± standard deviation (*x* ± SD). One-way ANOVA was used to compare the differences among groups, followed by the post hoc LSD method (homogeneity of variance) or Tamhane method (heterogeneity of variance). A *P* value of < 0.05 was considered statistically significant.

## Results

### Torque resistance and fibular rotation among the different groups under internal rotation and external rotation force

There were significant differences in torque resistance and fibular rotation angle between the intact and post-malreduction groups, and the foot was under internal load (torque 6.75 ± 1.14 vs. 5.29 ± 0.95 Nm, *t* = 2.412, *P* = 0.036; rotation angle − 1.79 ± 0.21 vs. − 2.27 ± 0.19 degree, *t* = 4.216, *P* = 0.002). In addition, a significant difference in torque resistance was found between the intact and ant-malreduction groups, while the foot was under external load (3.70 ± 0.57 vs. 3.02 ± 0.41 Nm, *t* = 2.380, *P* = 0.039). Fibular rotation was also detected to be significantly different between the intact and post-malreduction groups under both internal (− 1.79 ± 0.21 vs. − 2.27 ± 0.19 degree, *t* = 4.216, *P* = 0.002) and external (3.57 ± 0.62 vs. 4.84 ± 0.74 Nm, *t* = − 3.228, *P* = 0.017) loads (Table 1).

**Table 1:** Data of torque (Nm) and fibular rotation (degree) among the different groups under internal/external rotation load

Load model	Group	Torque	*t*	*P*	Rotation	*t*	*P*
Internal rotation	Intact	6.75 ± 1.14			− 1.79 ± 0.21		
	Anatomical	6.61 ± 0.99	0.239	0.816	− 1.87 ± 0.24	0.635	0.540
	Post-malreduction	5.29 ± 0.95	2.412	0.036*	− 2.27 ± 0.19	4.216	0.002*
	Ant-malreduction	6.13 ± 0.78	1.111	0.292	− 2.07 ± 0.22	1.111	0.292
External rotation	Intact	3.70 ± 0.57			3.57 ± 0.62		
	Anatomical	3.45 ± 0.37	0.818	0.432	3.82 ± 0.71	− 0.635	0.540
	Post-malreduction	3.22 ± 0.53	1.438	0.181	4.47 ± 0.45	− 2.869	0.017*
	Ant-malreduction	3.02 ± 0.41	2.380	0.039*	4.84 ± 0.74	− 3.228	0.009*

*Statistical significance: *P* < 0.05

### The 3D displacement of the syndesmotic incisura in the internal/external rotation model

#### Internal loading model

No differences were found among all groups for axial displacement (intact vs. anatomical, *P* = 0.430; intact vs. post-malreduction, *P* = 0.326; intact vs. ant-malreduction, *P* = 0.391; anatomical vs. post-malreduction, *P* = 0.842; anatomical vs. ant-malreduction, *P* = 0.944; post-malreduction vs. ant-malreduction, *P* = 0.897).

**Fig. 4:**
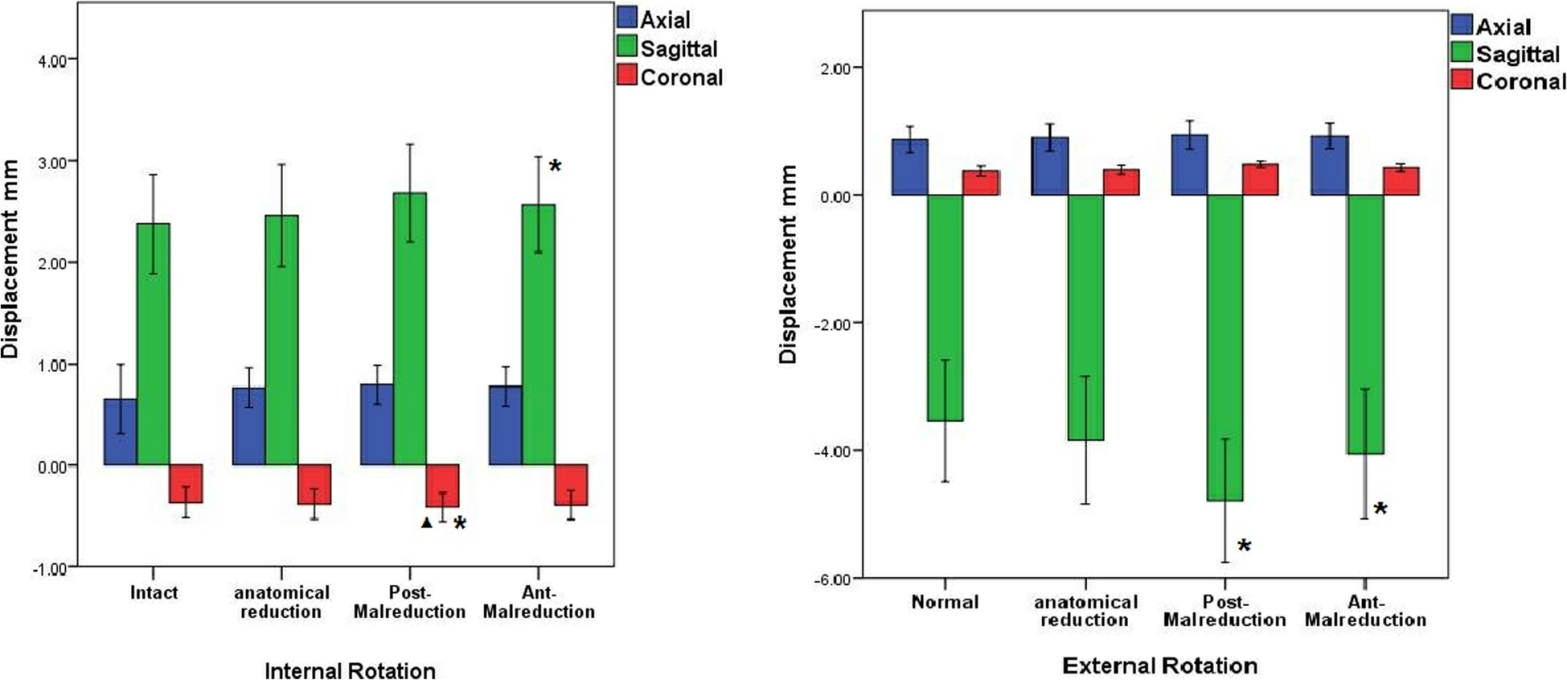
Summary of the sagittal, axial, and coronal displacements in the external and internal rotation models. Asterisk denotes the significant difference when compared with the intact group. Triangle denotes significant difference when compared with the anatomical group

There were significant differences in sagittal (anterior-posterior) displacement between the intact and posterior malreduction group (*P* = 0.011). Also, there were significant difference between anatomical and posterior malreduction group (*P* = 0.020) in sagittal displacement. However, there were no significant differences in the other groups (intact vs. anatomical, *P* = 0.787; intact vs. ant-malreduction, *P* = 0.302; anatomical vs. ant-malreduction, *P* = 0.441; post-malreduction vs. ant-malreduction, *P* = 0.099) (Fig. 4).

No differences were found among all groups for coronal (medial-lateral) displacement (intact vs. anatomical, *P* = 0.844; intact vs. post-malreduction, *P* = 0.597; intact vs. ant-malreduction, *P* = 0.768; anatomical vs. post-malreduction, *P* = 0.739; anatomical vs. ant-malreduction, *P* = 0.922; post-malreduction vs. ant-malreduction, *P* = 0.814).

#### External loading model

No differences were found among all groups for axial displacement (intact vs. anatomical, *P* = 0.797; intact vs. post-malreduction, *P* = 0.562; intact vs. ant-malreduction, *P* = 0.656; anatomical vs. post-malreduction, *P* = 0.745; anatomical vs. ant-malreduction, *P* = 0.850; post-malreduction vs. ant-malreduction, *P* = 0.892).

Merely the intact and anterior malreduction groups were significantly different in terms of sagittal displacement (intact vs. ant-malreduction, *P* = 0.034) (intact vs. anatomical, *P* = 0.598; intact vs. post-malreduction, *P* = 0.298; anatomical vs. post-malreduction, *P* = 0.600; anatomical vs. ant-malreduction, *P* = 0.098; post-malreduction vs. ant-malreduction, *P* = 0.243).

For coronal displacement, merely intact and posterior malreduction (*P* = 0.013), and anatomical and posterior malreduction (*P* = 0.038) were significantly different (intact vs. anatomical, *P* = 0.607; intact vs. ant-malreduction, *P* = 0.179; anatomical vs. ant-malreduction, *P* = 0.394; post-malreduction vs. ant-malreduction, *P* = 0.192) (Fig. 2).

## Discussion

The stabilizing structure of the distal syndesmosis includes the anterior and posterior inferior tibiofibular ligament, and interosseous membrane. Distal syndesmosis is an amphiarthrosis, which has a joint motion range of 2–5° on the coronal, sagittal, and horizontal planes [7, 8]. Although the distal tibiofibular syndesmosis has micro-motion, its stability is an important guarantee for normal weight bearing and function of the ankle. In our study, the rotation angle of syndesmosis (fibular relative to tibia) had significant change between intact group and malreduction group. It refers that malreduction does affect the stability of syndesmosis. Huber et al. [9] confirmed through biomechanical studies that screw fixation of the tibiofibular syndesmosis still has a joint motion range of 0.5–2.5°. Wang et al. [10] found that the fibula had an average rotation of approximately 1° relative to the tibia through 3D image analysis. In our biomechanical study, syndesmosis motion enlarged under malreduction condition that may also prove the overactivity had the high correlation of syndesmosis instability.

Studies have shown that when syndesmotic separation occurs, its displacement does not only shift in a single plane, but in both the sagittal and coronal planes [11]. If the rotation and anterior/posterior displacement of the fibula are not corrected, the fibula might be fixed in an improper position, leading to syndesmotic malreduction [3, 11, 12]. Moreover, due to the over-compression of the trans-syndesmotic screw, the tibiofibular space becomes too small. If judged simply from the position of the fibula and tibia, it is possible to misjudge the restoration distance [13, 14]. Through clinical follow-ups, it was found that syndesmotic malreduction is a factor that may affect ankle function [4, 15, 16]. However, it remains unclear whether this effect was due to the decline in ankle stability or the change in stress distribution of the contact area. Our study proved the obvious change when syndesmosis was fixed in a non-anatomical position. The torsional stress test under internal rotation conditions revealed that the torque was significantly smaller in the posterior malreduction group than in the intact group. However, the torque for the anterior malreduction group was relatively normal. In contrast, in the external rotation state, the torque was lower in the anterior malreduction group than in the intact group, while the torque in the posterior malreduction group was relatively normal. This also indicates that when the syndesmosis is not fixed at the anatomical position, its stability would decrease. Wei’s research revealed that when the syndesmosis was completely separated, the torque of the ankle increased by more than three times [17]. In addition, in 3D measurements, the external rotation stability of the tibiofibular syndesmosis was relatively weak, while the internal rotation was relatively stable [18]. However, in the external rotation state, the posterior malreduction (relative external rotation) was more stable than the anterior malreduction, while in the internal rotation state, the anterior malreduction was more stable. All these increasing relative motion of the syndesmosis indicate its stability decreased under the non-anatomical fixation. LaMothe et al. [19] demonstrated that the contact stress of the ankle joint was significantly increased after the ligaments were cut off, and the stress histogram revealed that the peak of the contact stress of the talus moved anteriorly and laterally. Moreover, in clinical practice, it was revealed that the unbalanced stress distribution was the main cause of ankle dysfunction and osteoarthritis [20].

The relative micro motion of syndesmosis was reported by many researchers. In Beumer’s biomechanical study [21], when the ankle is rotated internally or externally, the fibula has an inward or outward displacement of 0–2 mm relative to the tibia. When simulating partial weight bearing, the tibiofibular syndesmosis has a joint motion range of 3–5°. Rigby et al. [22] performed a 2-year postoperative follow-up on patients who had suture-button surgery and measured the tibiofibular clear space (TCS), tibiofibular overlap (TFO), and medial clear space (MCS). The results revealed that although fixed by suture-button, the relative position of syndesmosis had changed during follow-up time, and it revealed a certain trend of displacement after the joint fixation, and this kind of displacement was often observed in malreduction cases. In our study, regardless of the type of syndesmotic malreduction, its displacement along the axis of the lower extremity was not significantly different from that in the intact group. Furthermore, even if the fibula is fixed to the wrong position relative to the tibia, its axial stability is not affected either in the internal rotation stress state or external rotational stress state. Therefore, in the treatment of ankle fractures, it is critical to restore the length of the tibia and properly fix it. For sagittal and coronal stability, the mode of joint motion in different dimensions of the fibula relative to the incisura becomes the key to determine the joint stability of the syndesmosis. The further multi-dimensional displacement analysis of the syndesmosis revealed that under internal rotation conditions, the anteroposterior mobility of the posterior malreduction group (external rotation) increased, and the angle of rotation of the fibula with respect to the tibia was larger than that of the intact group, while under external rotation conditions, the position of the fibula relative to the incisura in the anterior malreduction group (internal rotation) significantly changed. Westermann et al. [23] reported that the syndesmosis had a certain degree of displacement after the suture-button fixation, and this malreduction had a tendency to automatically be reduced. Our study also confirms this finding: the internal rotation stability of the posterior malreduction was poor, indicating that the fibula is less resistant in the direction of the reversed deforming force, and has a greater degree of motion. This also indicates that the instability of syndesmotic malreduction might force the fibula to displace to the anatomic position with overwhelming deforming force, which could result in the excessive activity of the tibiofibular syndesmosis after fixation. Similarly, Teramoto [24] performed a 3D analysis on the joint instability of the syndesmotic separation and found that ankle external rotation instability could be caused by simple anterior tibial-fibular ligament damage. The above studies all revealed that under the condition that the syndesmosis was unstable, the mechanical state of the joint would be significantly changed, and the ankle function might be impaired.

The limitations of the study were as follows: in order to avoid the effects of multiple drilling holes, a 3D-printed guide plate was used to set different bone canals, and single suture-button fixation was used to minimize the measurement error between different specimens. Some studies have shown that using merely a single button cannot achieve complete stability of the syndesmosis [18]. However, in this study, the intact and anatomical groups had no significant differences in stability. Furthermore, there were also studies [25, 26] that revealed that the stability of the syndesmosis had no significant difference between suture-button fixation and screw fixation. Due to the limitation in sample size, the anatomy variation of the tibiofibular syndesmosis incisura was not considered [5].

## Conclusions

Malreduction of the syndesmosis does affect the stability of the syndesmotic fixation. No matter anterior or posterior malreduction of the fibular, the stability of torque resistance of the system was decreased under non-anatomical fixation. The relative motion of the syndesmosis under three-dimensional space was also significantly changed in malreduction group. The results of the study reveal the relationship between instability and syndesmosis malreduction and provide more biomechanical evidence on unsatisfied clinical outcome of ankle fracture fixation under syndesmotic malreduction conditions.

## Data Availability

The datasets generated and analyzed during this study are available from the corresponding author on reasonable request.

## Abbreviations

CT: Computed tomography
MCS: Medial clear space
TCS: Tibiofibular clear space
TFO: Tibiofibular overlap
